# Biomechanical analysis of porous and dense dental implant-supported bridges in healthy and osteoporotic bones

**DOI:** 10.1371/journal.pone.0329558

**Published:** 2025-09-23

**Authors:** Hassan Mehboob, Abdelhak Ouldyerou, Abdulsalam A. Al-Tamimi, Ali Mehboob, Imad Barsoum

**Affiliations:** 1 Department of Engineering Management, College of Engineering, Prince Sultan University, Riyadh, Saudi Arabia; 2 Department of Mechanical, Materials and Aerospace Engineering, West Virginia University, Morgantown, West Virginia, United States of America; 3 Department of Industrial Engineering, College of Engineering, King Saud University, Riyadh, Saudi Arabia; 4 Advanced Digital & Additive Manufacturing Center, Khalifa University, Abu Dhabi, United Arab Emirates; 5 Department of Mechanical and Nuclear Engineering, Khalifa University, Abu Dhabi, United Arab Emirates; 6 Department of Engineering Mechanics, Royal Institute of Technology – KTH, Stockholm, Sweden; Tecnológico de Monterrey, MEXICO

## Abstract

Despite the high success rate of crown bridge dental implants, peri-implant bone resorption remains a persistent major biomechanical issue. This study examines the impact of the implant’s Young’s modulus, implantation technique, and loading conditions on bone remodeling in the region of interest (ROI) for varying bone qualities. Three-dimensional finite element models of three-unit bridge crowns (TUB), implants, and cancellous and cortical bones were constructed using SolidWorks software. Four implantation scenarios, two bone health conditions (healthy and weak bone), and dense and effective porous implants were simulated under two loading conditions (200 N and gradually decreasing 100 N, 80 N, and 40 N), employing Abaqus software, for 16 cases (n = 16). A user’s subroutine was programmed using Python to estimate the iterative changes (46 simulations of each case) in bone density at peri-implant bone. The simulated results demonstrated that effective porous implants outperformed and showed higher Young’s moduli in the ROI compared to the dense implants when a single implant was implanted. However, when two implants were inserted simultaneously, the effective porous implant outperformed in the case of healthy bone only.

## 1. Introduction

Dental implants and fixed partial dentures (FPDs) are commonly used in prosthetic treatments, primarily to replace damaged or diseased teeth and restore their functionality [1,2]. These restorations may range from single-tooth implants to multi-unit bridgeworks. Despite their clinical success, the long-term performance of implants remains challenging due to the complex biomechanical forces acting on surrounding bones. Notably, FPDs anchored on implants can exhibit failure mechanically and biologically [3]. Despite their high survival rates (95% after 5 years and 86.7% after 10 years of follow-up), complications remain frequent, occurring in 38.7% of cases [4].

The success of prosthetic treatments depends on numerous factors such as implant design, bone condition, and biological and mechanical factors. After implantation, the implant is expected to osseointegrate completely with the surrounding bone to perform its function for a lifetime similar to the natural tooth. However, several complications may arise after dental implantation, including non-osseointegration due to surgical trauma or implant failure after complete osseointegration during the implant service life [5].

Conventional dental implants are typically fabricated from a dense titanium alloy (Ti), which possesses a significantly higher Young’s modulus (110 GPa) compared to that of the implant surrounding bone (ranging from 0.45 to 20 GPa). This high mismatch between the implant and bone properties causes an imbalance in the natural mechanical environment in the surrounding bone, often resulting in stress shielding and bone resorption. Also, porous implants facilitate bone ingrowth into the porous structure, establishing a mechanical interlocking at the bone-implant interface. Because of their porous geometry, the bone in-growth speeds up osseointegration and bone healing [6]. Moreover, porous implants reduce the volume of material for implant manufacturing, thus offering a lightweight structure for better patient comfort [7]. Therefore, reducing the implants’ stiffness to match the bone’s properties and maintain the biomechanical environment is one of the main concerns of the researchers, which can alleviate the stress shielding and bone resorption [8].

The stiffness of the Ti implants can be controlled during the design phase by introducing porous architecture and can be fabricated with the additive manufacturing (AM) technique [9]. AM possesses the capability to create complex geometries unattainable using conventional methods [10]. Rapid prototyping can be iterated multiple times until the component meets diverse requirements, including user preferences, cost efficiency, and regulatory compliance. Consequently, there has been an emphasis on pioneering research initiatives that utilize additive manufacturing methods to produce finished items [11,12]. Another advantage of additive manufacturing is its enhancement of the manufacturing process, coupled with a reduction in production costs, carbon emissions, energy consumption, and material wastage [13]. These porous architectures are easily digitally modelled with computer-aided design (CAD). Various fascinating bio-inspired designs of implants can be modelled and simulated with software to assess their biomechanical performance. Finite element analysis (FEA) can efficiently simulate various conditions such as masticating forces, bone quality, implant design, and bone-implant interfacial contact (e.g., thread design and dental cavities), which are less laborious, cost-effective, and time-consuming compared to *in vitro* or *in vivo* experiments [14,15].

Different theories have defined criteria to estimate the changes in bone densities around the implants. These theories are programmed in the user’s subroutines and coupled with FEA to estimate the changes in the bone under certain simulated conditions. Frost presented the mechanostat hypothesis to refine Wolff’s law [16], which is based on the level of strains produced in the bone. The mechanostat hypothesis divides bone remodelling into four major zones: disuse, physiological, overuse, and pathological overload. Several FE studies have examined bone responses within single dental implants using the mechanostat hypothesis [17–20]. Another theory presents bone remodelling based on the level of strain energy density (SED) in the surrounding bone [21].

Recent finite element studies have examined bone remodelling within dental implants using SED theory. Rajaeirad et al. [21] studied the potential for osseointegration and remodelling of a single dental implant made from Titanium-Hydroxyapatite functionally graded material varying volume fractions 0–20%. The results revealed that peri-implant bone density was improved by using a functionally graded material implant. Rungsiyakull et al. [22] explored the effect of bone quality and loading patterns on the remodelling process of one single implant. The results revealed that higher stresses and bone remodeling were observed when off-axial load was used on the implant. Poovarodom et al. [23] evaluated the influence of subcrestal implant placement depth on bone remodelling. Their findings showed that a higher bone density was achieved with a deeper implant placement. Rungsiyakull et al. [24] investigated bone remodelling responses of implant-implant-supported and tooth-implant-supported FPDs. The bone remodeling analyses revealed that the tooth-implant scheme offers greater osseointegration. Field et al. [25] studied bone remodelling of two prosthetic configurations, inlay and onlay FPDs. Their study found that both FPD designs showed similar bone densities, with a more uniform bone density distribution in the onlay FPD. Wang et al. [26] simulated the bone biomechanical response, with and without cantilever two-unit FPDs. The results showed higher bone resorption in the cortical bone in the case of the cantilever FPD compared to the other case. The aforementioned studies have shortcomings in investigating the effect of implant stiffness on bone remodeling in healthy and weak bones in the case of FPDs with their insertion sequence. Moreover, the incorporation of mechanostat theory with SED theory has the potential to investigate the excessive level of strains and related bone resorption.

This study investigates the bone remodelling at the peri-implant bone when dense and effective porous implants were fixed with a TUB in healthy and weak bones. Three-dimensional (3D) finite element models (FEM) of mandible cortical and cancellous bones were constructed. Masticating forces were used to simulate the bone remodelling around the implants. A bone remodelling algorithm based on mechanostat and SED was programmed in the user’s subroutine using Python, and iterative calculations were performed to estimate the changes in bone densities. The null hypothesis was that dense and effective porous implants with TUB and their inserting sequences would not affect the biomechanical environment and thus would not affect the bone density around the implants.

## 2. Materials and methods

### 2.1. Geometric acquisition of 3D parts

**Fig 1:**
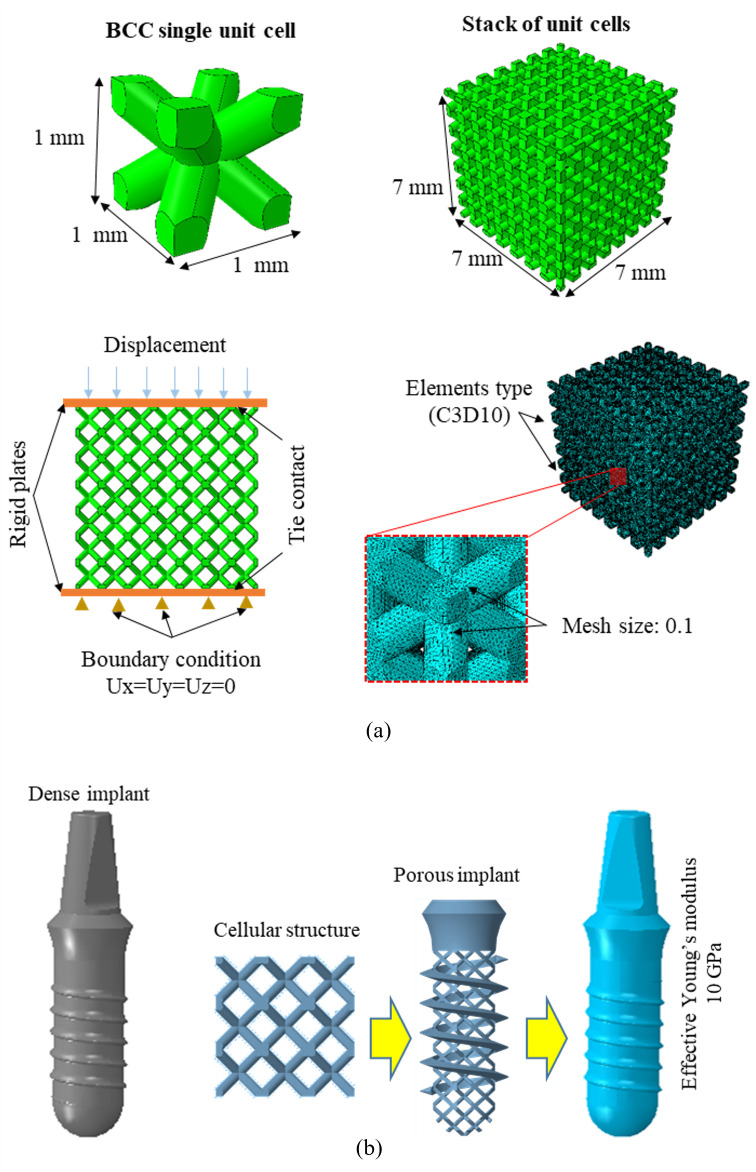
Designing of porous implant to solid implant; (a) compressive simulation of porous BCC structure to obtain effective material properties, and (b) Dense and porous implant with effective material properties.

**Fig 2:**
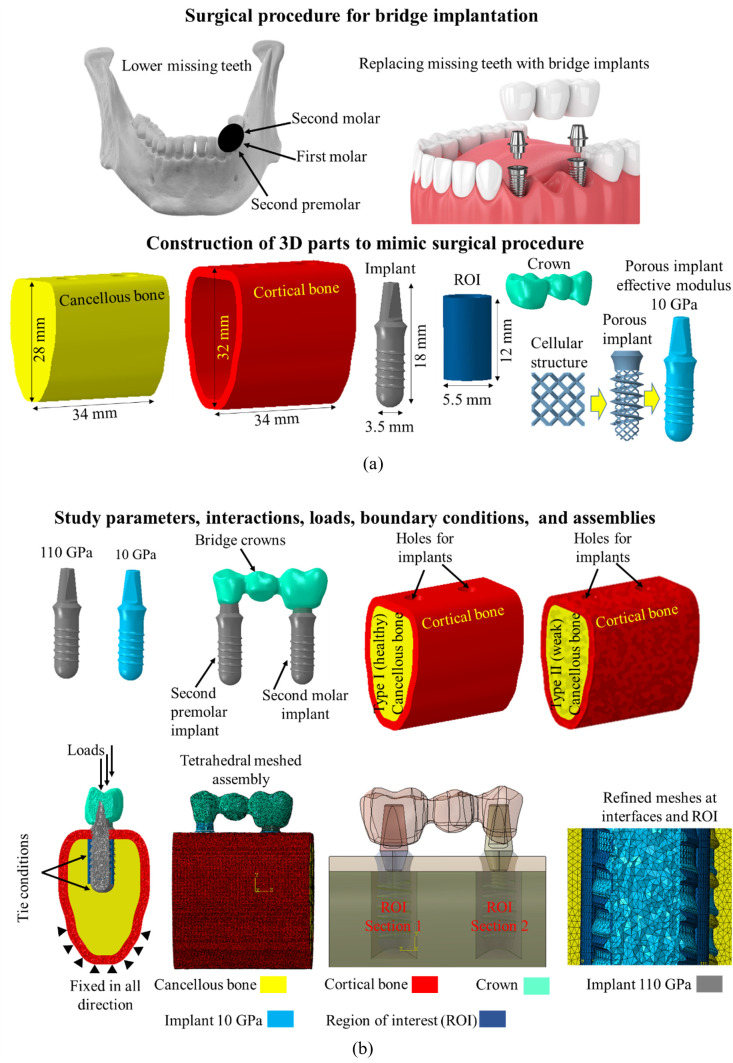
Finite element modeling and analysis of mandible implants; (a) dimensions of modeling of bones, bride crowns, implants, and region of interest, and (b) design parameters, meshes, elements, interactions, loadings, and boundary conditions.

The body-centered cubic (BCC) structure includes a higher rate of cell proliferation compared to other structures, which makes it suitable for high surface area and interconnected pores that are beneficial for cell attachment, cell growth, and nutrient transport [27]. Thus, the finite element model of BCC structure was modelled in SolidWorks^©^ (Dassault Systèmes Solid Works© Corp., Concord, USA) and stacked to simulate the compressive properties using Abaqus 2017 (Dassault Systemes, France, Abaqus), as shown in Fig 1a. The strut thickness of the BCC structure was set to 1 mm which yielded a porosity of 59%. The effective Young’s modulus was determined from the compression test simulation and listed in Table 1. The lower part of the implant was assumed porous and given effective properties of porous BCC structures as shown in Fig 1b. 3D FEM of cancellous and cortical bones, and TUB structure were constructed using SolidWorks^©^ (Dassault Systèmes Solid Works© Corp., Concord, USA), as shown in Fig 2a. The geometry of the lower section of mandible was taken which included second premolar, first molar, and second molar regions. Cancellous bone was enveloped by cortical bone with a thickness of 2 mm [28]. The heights of the cancellous bone and cortical bone were 28 mm and 32 mm, respectively. The length of the bridge was 19 mm with diameters of 6 mm at the second premolar crown and 8 mm at the second molar crown. The height of the implant was 18 mm with a diameter of 3.5 mm. For computational purposes, the effective porous implant was modelled as a porous cellular structure, which was simplified into a dense structure with the obtained effective material properties equivalent to those of the porous structure, as shown in Fig 2a.

**Table 1:** Material properties used in finite element analysis.

Material	Young’s modulus (E, GPa)	Poisson’s ratio (υ)	Density (ρ, g/cm^3^)
**Bulk titanium alloy (E110)**	110	0.3	–
**Porous titanium (E10, effective Young’s modulus)**	10	0.3	–
**Crown**	40	0.3	–
**Cortical bone**	14	0.3	–
***Cancellous bone***			
**Healthy bone (P0.7)**	1.3	0.3	0.7
**Weak bone (P0.345)**	0.345	0.3	0.45

### 2.2. Material properties of parts

Abaqus was used to simulate the effects of implants with different bone conditions. Linear elastic properties were assigned to the cancellous and cortical bones as listed in Table 1 [29–33]. Cortical and cancellous bones were assigned two types of material properties to mimic healthy bone P0.7 (Young’s modulus 1.3 GPa, and bone density 0.7 g/cm^3^) and weak bone P0.45 (Young’s modulus 0.345 GPa, and density 0.45 g/cm^3^). The implants were assigned with the obtained effective Young’s modulus of porous structure (10 GPa, E10) and Young’s modulus of dense Ti (110 GPa, E110). All crowns of the bridge were assigned with Young’s modulus of 80 GPa, as shown in Table 1. The Young’s modulus (*E*) of the cancellous bone and apparent density were calculated using Eq. 1, which was suggested by Carter and Hayes [34,35].


$$E=C\rho^{3}$$ (1)

where ρ is the apparent density of the cancellous bone in g/cm^3^ and *C* is 3790 MPa.cm^9^/g^3^. The Young’s modulus of the cancellous bone was calculated for each element of the region of interest (ROI).

### 2.3. Assembly scenarios of TUB

**Fig 3:**
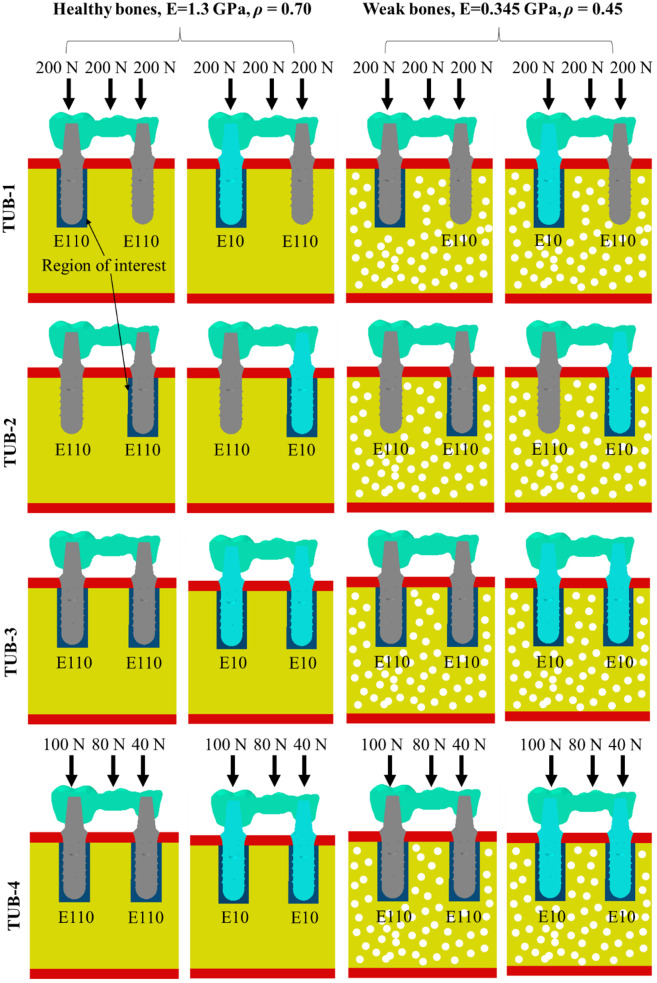
A detailed graphical presentation of the fixation technique of different implants, regions of interest, and bone health.

Two implants were inserted at the position of the second premolar and second molar teeth to prepare a TUB using Abaqus, as shown in Fig 2b. Four different TUB implantation scenarios were created in the bone segments. All implants were assumed to be fully osseointegrated [36] with cancellous bones, and bone remodelling (changes in bone densities) was estimated under different conditions. The ROI (106 mm^2^) for bone remodelling was defined as a cylindrical area around the implant (navy blue color around implant screw), as illustrated in Fig 3. The fixation of the implants E110 (grey color) and E10 (baby blue color) are also shown in Fig 3. The left side implants represent the second molar tooth and the right side implants represent the second premolar tooth. In the first scenario TUB-1, one implant at the second premolar position was fixed assuming an equilibrium condition (without bone remodelling), and a second implant was fixed in the bone at a second molar position to estimate bone remodelling in ROI and vice versa in TUB-2 second scenario (i.e., estimate bone remodeling in the second premolar). In the third and fourth scenarios (TUB-3 and TUB-4), implants were fixed at the second premolar and second molar positions to simultaneously evaluate bone remodelling in both implants within the ROI, with varying loading conditions, which will be detailed in the next section.

### 2.4. Interactions, mesh, loads, and boundary conditions

The implants were tied with the cancellous and cortical bones in Abaqus to mimic the full osseointegration conditions, assuming that the healing process was completed successfully. The crowns were also tied with implants to mimic the bonded condition. The assemblies were meshed with variable sizes (with coarser mesh applied in areas distant from the ROI and finer mesh within the ROI) using quadratic tetrahedral C3D10 elements as shown in Fig 2b. An appropriate mesh size (0.2 mm) was selected after mesh sensitivity analysis (stresses in ROI were checked with 0.2, 0.3, 0.4, and 0.5 mm mesh sizes) to ensure the accuracy of the results. In the first three TUB implantation scenarios, the crowns were loaded with masticating axial forces of 200 N at each molar crown. In the fourth TUB-4 implantation scenario, gradually increasing loads were applied; 40 N at the second premolar, 80 N at the first molar, and 100 N at the second molar crown [37], as shown in Fig 3. The sides of the bones were fixed in all directions to avoid movement and rotations in any direction (Fig 2b).

### 2.5. Finite element analysis coupled with bone remodelling

The four TUB implantation scenarios, depicted in Fig 3 and detailed in Table 2, represent different configurations and loading conditions. Two implant stiffness (10 GPa (E10) and 110 GPa (E110)) [38], and two bone health conditions (Young’s modulus 0.345 GPa and bone density 0.45 g/cm^3^ (P0.45), and Young’s modulus 1.3 GPa and bone density 0.7 g/cm^3^ (P0.7)) were simulated using Abaqus, resulting in a total of 16 cases (4 × 2 × 2 = 16 simulations), outlined in Table 3. The bone remodelling was estimated in ROIs in all cases. The bone remodelling requires biomechanical responses from the ROI produced by applied masticating forces. These biomechanical responses cause changes in the bone density around the implant. The studies showed that the elastic SED (*U* in J/cm^3^) is a widely acceptable biomechanical response to predict the changes in bone density [39] and is calculated according to Eq. (2).

**Table 2:** Implantation scenarios with bone health, implant type, ROI and loading conditions.

Implantation Scenarios	Cancellous bone		Implant		Region of interest (ROI)	Region of interest (ROI) position	Loading (N)
**TUB-FPD-1**	P0.7	P0.45	E10	E110	1	Second molar	200 N on each molar crown
**TUB-FPD-2**	P0.7	P0.45	E10	E110	1	Second premolar	200 N on each molar crown
**TUB-FPD-3**	P0.7	P0.45	E10	E110	2	Second molar and second premolar	200 N on each molar crown
**TUB-FPD-4**	P0.7	P0.45	E10	E110	2	Second molar and second premolar	40 N at the second premolar, 80 N at the first molar, and 100 N at the second molar crown

**Table 3:** Simulation runs for all parameters including bone, implant, ROI, and loading conditions.

Simulation	Cancellous bone	Implant		Region of interest (ROI)		Loading condition (N)		
		Second molar	Second premolar	Second molar	Second premolar	Second premolar	First molar	Second molar
1	P0.7	E110	E110	Yes	No	200	200	200
2	P0.7	E10	E110	Yes	No	200	200	200
3	P0.7	E110	E110	No	Yes	200	200	200
4	P0.7	E110	E10	No	Yes	200	200	200
5	P0.7	E110	E110	Yes	Yes	200	200	200
6	P0.7	E10	E10	Yes	Yes	200	200	200
7	P0.7	E110	E110	Yes	Yes	200	200	200
8	P0.7	E10	E10	Yes	Yes	200	200	200
9	P0.45	E110	E110	Yes	No	200	200	200
10	P0.45	E10	E110	Yes	No	200	200	200
11	P0.45	E110	E110	No	Yes	200	200	200
12	P0.45	E110	E10	No	Yes	200	200	200
13	P0.45	E110	E110	Yes	Yes	40	80	100
14	P0.45	E10	E10	Yes	Yes	40	80	100
15	P0.45	E110	E110	Yes	Yes	40	80	100
16	P0.45	E10	E10	Yes	Yes	40	80	100


$$U=\frac{1}{2}\sigma_{ijk}\varepsilon_{ijk}$$ (2)

where $\sigma_{ijk}$ and $\varepsilon_{ijk}$ are the stresses and strains in the x, y, and z directions of each element, respectively. The value of *U* determines the level of biomechanical stimulus according to the bone density in each element using Eq. (3) [40].


$$\Xi =\frac{U}{\rho }$$ (3)

where Ξ is the biomechanical stimulus (J/g) and ρ is the density of the bone (g/cm^3^).

**Fig 4:**
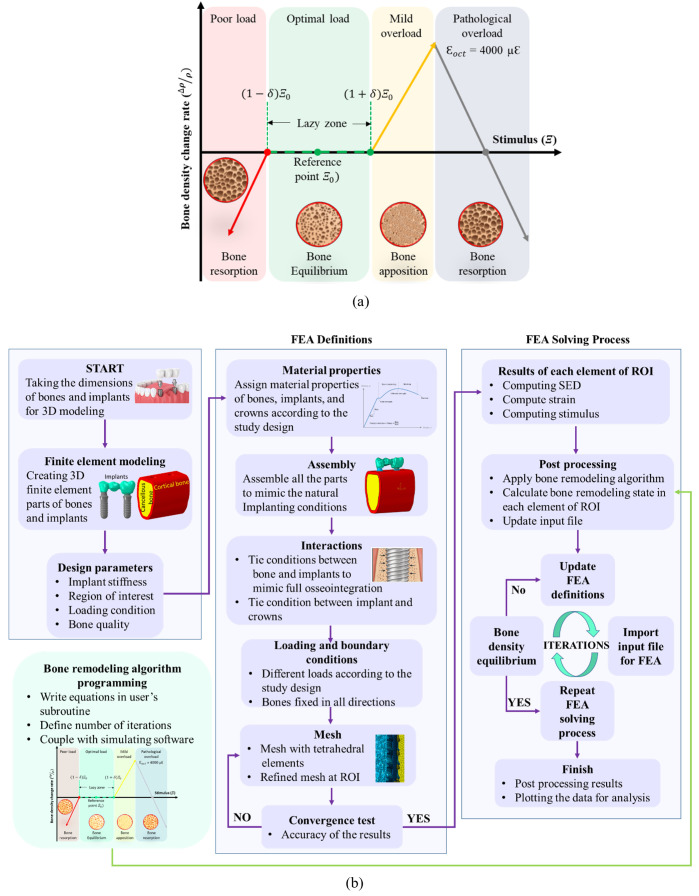
A detailed process of iterative simulation coupled with bone density algorithm; (a) a function of stimulus and related bone density changes, and (b) iterative calculations of finite element analysis coupled with user’s subroutine for changes in bone density.


The bone remodelling algorithm estimates the changes in bone density according to the biomechanical stimulus and the threshold value of biomechanical stimulus $\Xi_{o}$. Fig 4a shows the bone density change rate within three regions: the reduction in bone density (bone resorption), no changes in bone density (lazy zone), and an increase in bone density (bone apposition). However, at a higher level of stimulus, the bone apposition does not occur, and instead, bone resorption starts; hence, a threshold value was defined to reduce the bone density according to the mechanostat principle to mimic the pathological overload condition. The value of pathological overload stimulus was > 4000 µε [41]. The pathological overload value was calculated by octahedral shear strain ($\varepsilon_{oct}$in µε), representing the most appropriate strain for the mechanostat theory [42,43], as shown in Eq. (4). Hence, a fourth region was incorporated into Fig 4a as bone resorption under pathological overload.

$$\varepsilon_{oct}=\frac{2}{3}\sqrt{(\varepsilon_{1}-\varepsilon_{2}{)}^{2}+(\varepsilon_{2}-\varepsilon_{3}{)}^{2}+(\varepsilon_{3}-\varepsilon_{1}{)}^{2}}$$ (4)

where $\varepsilon_{1}$, $\varepsilon_{2}$, and $\varepsilon_{3}$ are principal strains.

The change in the bone density (Δρ) was estimated in each iteration using Eqs. (5) [44].


$$\Delta \rho =\left\{ \begin{array}{c} \left(\Xi -(1-\delta )K\right)Aboneresorption\Xi <(1-\delta )K\\ 0lazyzone(1-\delta )K<\Xi <(1+\delta )K\\ \left(\Xi -(1+\delta )K\right)Aboneapposition\Xi_{u}>\Xi >(1+\delta )K\\ \left(\Xi -(1+\delta )K\right)\text{A}-{\left(\Xi -(1+\delta )K\right)}^{2}\text{B}boneresorption\varepsilon_{oct}>4000\mu \epsilon \end{array}\right.$$ (5)

where δ,K, $\Xi_{u}$, A, B, and are the bandwidth of the lazy zone (10%) [45], the reference value of the stimulus 0.000036 J/g/cm^3^ according to the difference in cross-section areas of the mandible and femur [46], the upper limit of the stimulus, the bone remodelling rate constant 60 month.g/cm^5^ for bone apposition and 120 month.g/cm^5^ for bone resorption [46,47].

The bone remodelling algorithm was programmed in the Python subroutine, coupled with Abaqus. Iterative calculations (see **Fig 4b**) were carried out to perform the finite element simulations for the aforementioned conditions. Initially, at time step 0, the cancellous bone densities were modelled as homogeneous materials. As the masticating loads were applied to the bridge, the stress and strain were reported for each element of cancellous bone (ROI) in x, y, and z directions to calculate the SED as indicated in Eq. 2. Then the stimulus value was calculated using Eq. 3 for each element of cancellous bone in ROI using the SED and the current bone density of that element in iteration *n*. Meanwhile, octahedral shear strain using Eq. 4 was also calculated in each element of cancellous bone (ROI) using the strain values to check if the bone is pathologically overloaded. Finally, Eq. 5 was employed to calculate the changes in the apparent bone density Δρ in each element of cancellous bone (ROI). The values of the Δρ of each element were used to update the density to calculate the Young’s modulus of each element of cancellous bone (ROI) using Eq. 1. This iterative process continued till the overall equilibrium of changes in cancellous bone density (ROI) by < 0.04% was achieved, as shown in Fig 4b.

## 3. Results and discussion

Finite element models of mandibular bone (cortical and cancellous) sections were constructed in SolidWorks. Two holes were created in the bones to insert metallic implants. One implant was modeled as dense titanium, and the other as effective porous titanium (effective elastic modulus of 10 GPa). Both implants were fixed in a bone section. A TUB was also modelled to be fitted with implants to restore the functionality of natural teeth. Masticatory forces were applied to the bridge. Regions of interest were defined at the peri-implant bone section to evaluate the biomechanical performance of implants closely.

### 3.1. Strain energy density of ROIs

**Fig 5:**
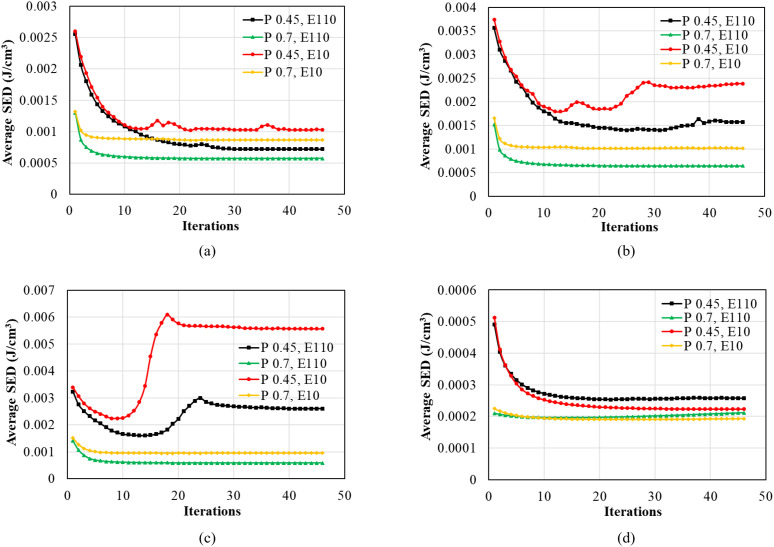
Average strain energy density (SED) in the region of interest (ROI) calculated using iterative calculations of finite element analysis; (a) TUB-1, (b) TUB-2, (c) TUB-3, and (d) TUB-4.

Fig 5 shows the average SED, J/cm^3^, in ROIs calculated in each iteration. Since stimulus is a function of SED that can control the changes in bone density [48]. The stresses and strains are mostly presented around the implants; thus, ROI was defined around the implants to investigate the changes in bone density [49,50]. Fig 5a presents the SED in ROI 1 in the case of TUB-1, when dense and effective porous implants were inserted in healthy and weak bones. It was observed that SED in weak bones was initially higher, 0.00255 J/cm^3^ for dense implants and 0.0026 J/cm^3^ for effective porous implants; however, a drastic decrease was seen in the first 10 iterations, reaching a level of 0.00108 J/cm^3^ and 0.0011 J/cm^3^, respectively. On the other hand, in the healthy bones, a different trend was seen; the initial level of SED was lower in dense and effective porous implants, starting from 0.0012 J/cm^3^ and 0.0013 J/cm^3^, respectively. A sharp decline was within the first 4 iterations, reaching 0.00068 J/cm^3^ and 0.0009 J/cm^3^ for dense and effective porous implants, respectively, and the rest of the iterations showed a slight decrease in SED until stability. Similarly, Fig 5b shows the SEDs calculated in the case of TUB-2 when dense and effective porous implants were inserted in healthy and weak bones. The trend of SED in the case of TUB-2 is similar to the case of TUB-1. The SED in the weak bone was 0.0035 J/cm^3^ for effective porous implants and 0.0031 J/cm^3^ when dense implants were inserted, and the loads were applied. Again, a sharp decline is observed in the first 10 iterations and stabilizes for the rest of the iterations, similar to the case of TUB-1. In the case of healthy bone, the slight decrease of SED was just in the first few iterations and stabilized afterward. In the case of TUB-3 presented in Fig 5c, dense and effective porous implants in weak bones exhibited a decreasing trend in SED over the first 10 iterations. Subsequently, the SED followed a sharp rise between 10–20 iterations, peaking at 0.0028 J/cm^3^ for dense implants and 0.006 J/cm^3^ for effective porous implants, before stabilizing. Conversely, the dense and effective porous implants in healthy bone showed a similar trend in the cases TUB-1 and TUB-2. Fig 5d illustrates the case TUB-4, where the levels of initial SED were significantly lower than in other scenarios due to the considered low loads, despite an implantation scenario identical to the TUB-3 case. Weak bones with dense and porous effective implants presented slightly higher SED levels compared to healthy bone conditions. A sharp decline occurred during the first 10 iterations, after which the SED reached steadiness. A common trend was observed in dense and effective porous implants, where SED values were lower in the dense implants due to the difference in Young’s modulus and implant types [51–53]. Moreover, the results indicated that SED levels were influenced by bone health conditions, with weak bones demonstrating higher values than healthy bones. Consequently, the levels of SED were different, resulting in different bone remodeling around the implants [54,55].

### 3.2. Average stimulus of ROIs

**Fig 6:**
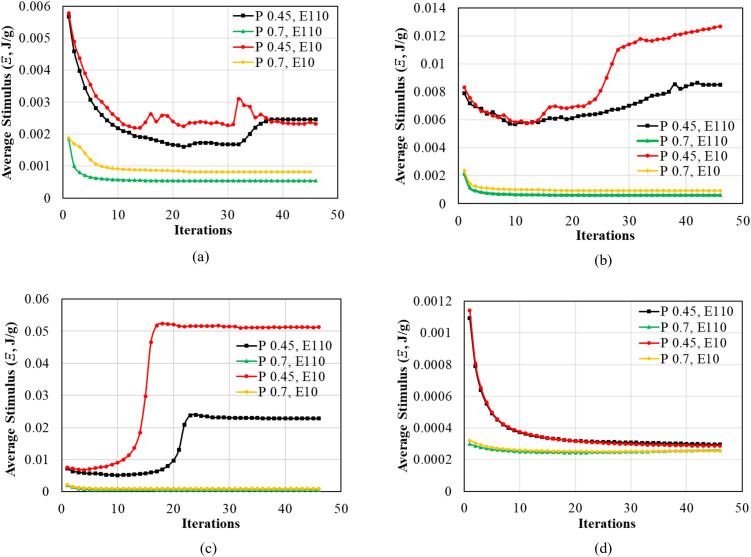
Average Stimulus (Ξ) in the region of interest (ROI) calculated using iterative calculations of finite element analysis; (a) TUB-1, (b) TUB-2, (c) TUB-3, and (d) TUB-4.

The stimulus (Ξ, J/g) is a ratio of SED and the bone density of iteration *n*, as described in Eq. 3. Thus, the values of the stimulus across all iterations were computed, as shown in Fig 6. Figs 6a-6b show the values of average stimulus in ROI in each iteration for the cases of TUB-1, TUB-2, TUB-3, and TUB-4, respectively. The stimulus values around the implant are the function of the SED generated in the ROI [35]. Therefore, the observed trends of the stimuli were identical to the obtained SED levels. For example, the stimulus of the TUB-1 case was higher in weak bones when dense and effective porous implants were fixed. However, a steep decline of the stimuli was noted after the first ten iterations, from 0.0056 J/g to 0.0021 J/g for dense implants and from 0.0057 J/g to 0.0024 J/g for effective porous implants, due to a decline in the SED. Eventually, the values stabilized, indicating bone density convergence in the ROI. This was similar to the remaining cases (i.e., comparing Figs 6b-6d to Figs 5b-5d). These values of stimulus determine the average bone density and corresponding Young’s modulus in peri-implant bone according to the quality of the bone, loading conditions, and type of implants [56].

### 3.3. Average young’s modulus of ROIs

**Fig 7:**
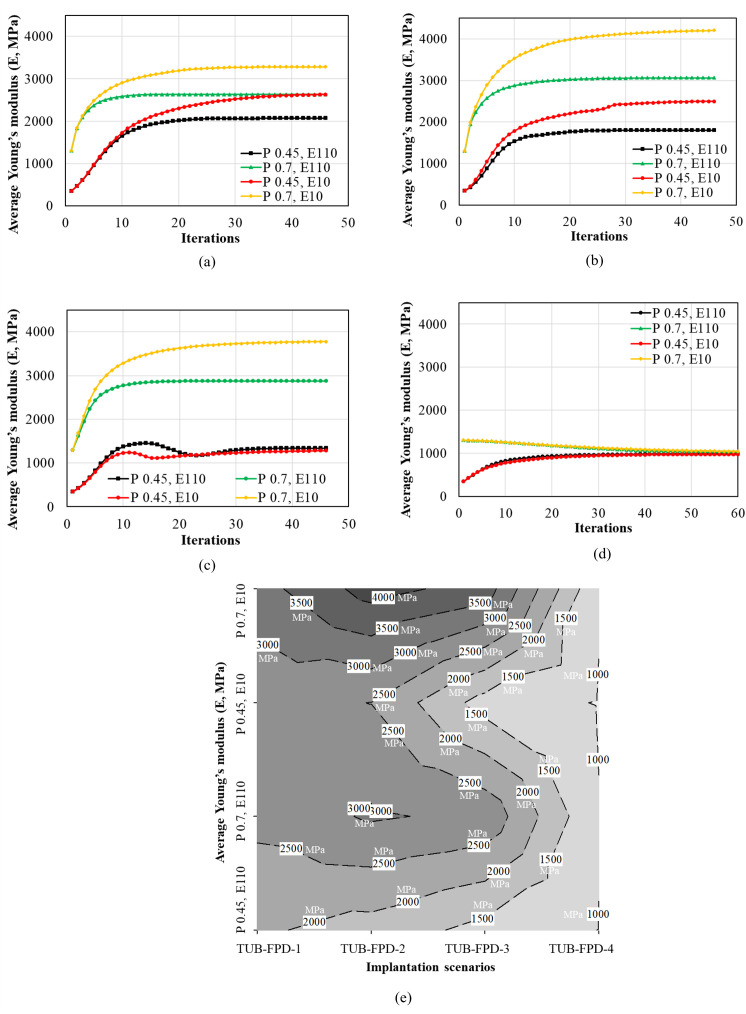
Average Young’s modulus (E) in the region of interest (ROI) calculated using iterative calculations of finite element analysis; (a) TUB-1, (b) TUB-2, (c) TUB-3, (d) TUB-4, and (e) contour plot showing average Young’s moduli (MPa) of all the simulated cases.

Fig 7 presents the average Young’s moduli in all the cases, TUB-1, TUB-2, TUB-3, and TUB-4. The Young’s modulus in the ROI for scenarios TUB-1 and TUB-2 (Fig 7a and 7b) increased significantly more in weak bones compared to healthy bones. In contrast, TUB-3 and TUB-4 (Figs 7c and 7d) implantation scenarios presented opposite results. In all scenarios, the remodelling process yielded the highest Young’s modulus in healthy bones fixed with effective porous implants, followed by healthy bones fixed with dense implants. For example, an average Young’s modulus of 3.28 GPa was achieved in the TUB-1 case, considering healthy bone using the effective porous implant. This demonstrated an increase in bone density, consistent with the bone density change law. However, using a dense implant in a healthy bone also increases bone density. It resulted in a lower average Young’s modulus of 2.63 GPa. A similar Young’s modulus value was achieved in weak bones with effective porous implants (2.63 GPa), while the lowest Young’s modulus was observed when considering weak bones with dense implants (2.07 GPa). In the case of TUB-2 (Fig 7b), the trend was similar to that of TUB-1, although with a higher average Young’s modulus in healthy bones. In the TUB-3 case (Fig 7c), where both implants were inserted together and a load of 200 N was applied [14], an increase in bone density and Young’s modulus was observed due to bone apposition. However, fixing using both implants simultaneously is not favorable in weak bones, as they exhibit a low Young’s modulus [57]. On the other hand, in the TUB-4 scenario (see Fig 7d), at the final iteration, all conditions resulted in the same average Young’s modulus. In this case, both implants were inserted simultaneously, and a gradually increasing load was applied to the crowns. This led to a decrease in bone density in healthy bones fixed with dense and effective porous implants because of the bone resorption mechanism. This can also be attributed to a lower level of stimuli caused by poor loading conditions. However, weak bones did not experience the same effect. Due to weak bone’s lower Young’s modulus, this lower level of loads on the crowns (40 N at the second premolar, 80 N at the first molar, and 100 N at the second molar crown) was able to stimulate the surrounding bone and slightly increase the average Young’s modulus of bone in ROI. The provided results corroborate with literature where different types of implants and geometry, along with varying loading conditions, generate distinct biomechanical environments around the peri-implant bone, leading to different bone remodelling and resulting in varying Young’s moduli of the surrounding bone [58–61].

Furthermore, a contour plot was generated to show the overall average Young’s moduli (MPa) across all the simulated cases as illustrated in Fig 7e. The plot shows the gradient of average Young’s modulus of bone is a function of the implantation technique (TUB-1, TUB-2, TUB-3, and TUB-4), bone quality, and implant stiffness. The maximum average Young’s modulus (dark grey color) was achieved when a healthy bone was implanted with an implant with an effective porous implant (effective Young’s modulus of 10 GPa) as presented in the TUB-2 case. Whereas the minimum average Young’s modulus of bone (light grey color) was noted in the case of poor loading (40 N at the second premolar, 80 N at the first molar, and 100 N at the second molar crown) when two implants were inserted simultaneously (TUB-4). These Young’s moduli reach to equilibrium state after certain iterations, and this state depends on various biomechanical factors generated in the surrounding bone [35,60].

### 3.4. Spatial distribution of the bone density in ROIs

**Fig 8:**
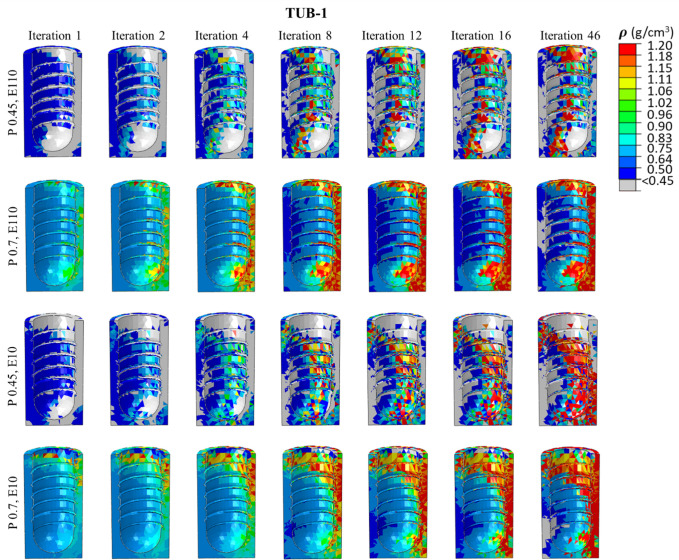
A spatial distribution of changes in bone density in the region of interest (ROI) in the case of TUB-1.

**Fig 9:**
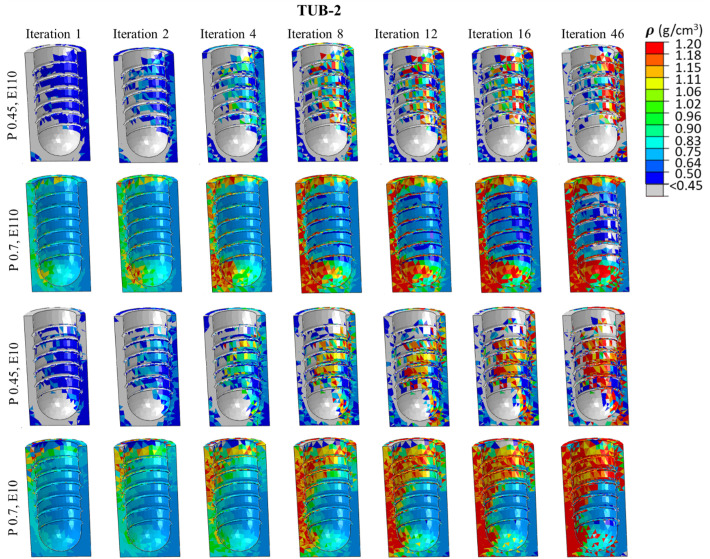
A spatial distribution of changes in bone density in the region of interest (ROI) in the case of TUB-2.

**Fig 10:**
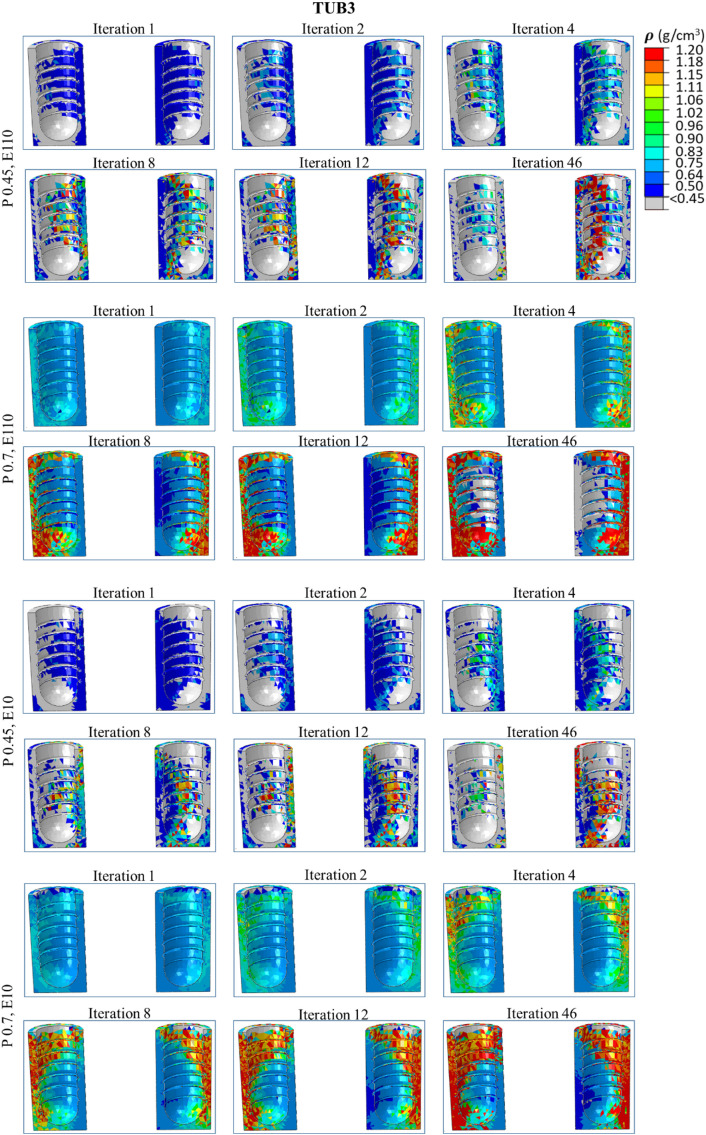
A spatial distribution of changes in bone density in the region of interest (ROI) in the case of TUB-3.

**Fig 11:**
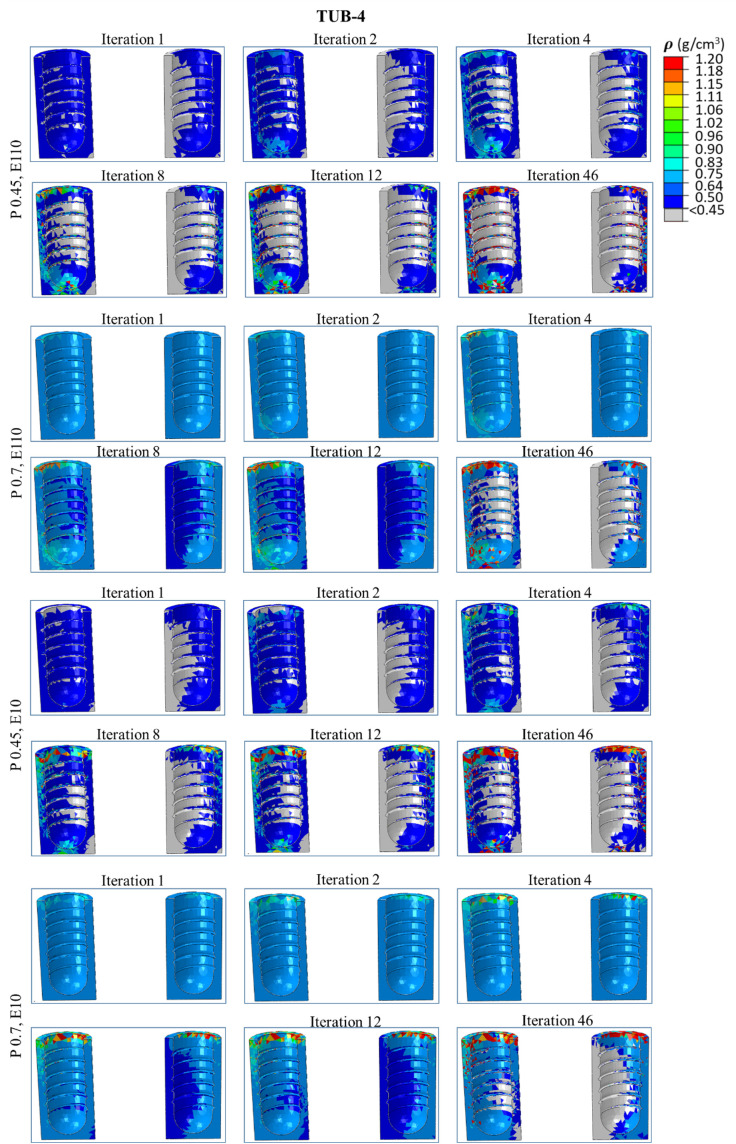
A spatial distribution of changes in bone density in the region of interest (ROI) in the case of TUB-4.

Figs 8**−**11 present nephograms of the qualitative change of the spatial distribution of the bone density (ρ) in each element of ROI in all cases where the change in Young’s modulus is a function of bone density [60]. The simulation initiated with the ROIs assigned an initial bone density (ρ) in all the elements of 0.7 g/cm^3^ for healthy bones and 0.45 g/cm^3^ for weak bones. During iterative calculations (iterations 1−46), bone density was changed based on the stimulus levels in different elements and regulated across iterations until equilibrium was achieved. Due to the complex shape of the bone-implant assembly, varying biomechanical stimuli are generated in the bone along the anterior, posterior, medial, and lateral directions, yielding different bone densities. The healthy bone was shown in light blue color, and the weak bone was in dark blue, as reflected in the density gradient legend in Fig 8. The results of the change in bone densities of 46 iterations are presented in Figs 8**−**11. The last iteration shows a mixture of resorbed bone in grey, apposition bone in red, and the bone with its original density. Comparing dense and effective porous implants in healthy and weak bones, the effective porous implant generated denser bone (red, orange, and yellow colors) compared to the dense implant in all scenarios. This is related to Young’s modulus, where less stiff implants generate higher stress on the bone, promoting greater bone apposition. The amount of bone resorption and apposition is related to the Young’s modulus, as shown in the average Young’s modulus (Figs 7a**-**7d), and to the bone density change. For example, Fig 10 shows the spatial distribution of bone density for the case TUB-3, where effective porous implant in weak bones showed a lesser amount of high density which corresponds to a lower average Young’s modulus presented in Fig 7c. This could be because of pathological overload when two effective porous implants were inserted simultaneously, which leads to the bone-to-bone resorption. In this case, dense implants are a better choice compared to effective porous implants. However, in healthy bone, effective porous implants can be implanted simultaneously, which relatively shows higher bone density and average Young’s modulus. On the other hand, the TUB-4 scenario (Fig 11) showed minimal differences in bone density between effective porous and dense implants, as they were nearly equivalent to each other, aligning with the average Young’s modulus (Fig 7d). This was due to a lower level of loads and to the poor loading where bone resorption was seen in most of the ROI region [62]. In contrast, in weak bone, the insertion of dense and effective porous implants leads to the formation of small islands of higher bone density, while most ROIs are dominated by bone resorption [55]. Similarly, comparing dense and effective porous implants in healthy bone in the case of TUB-1, the effective porous implant showed a denser bone (red, orange, and yellow colors), overall showing the cross-sectional view of the ROI. In the case of TUB-1, healthy and weak bones with effective porous implants show the maximum red and orange colors (a sign of higher bone density) with the least bone resorption in iteration 46 compared to the dense implants. This is evidence of having a higher average Young’s modulus of ROI as shown in Fig 7a. Similarly, in the case of TUB-2 in Fig 9, a higher bone resorption was observed (in grey color) in iteration 46 in healthy and weak bones with dense implants compared to effective porous implants. Bigger red, orange, and yellow islands were seen in effective porous implants, as shown in Fig 10 and Fig 7c, respectively. Fig 11 presents the implantation of two implants simultaneously, and a gradually increasing load (40 N at the second premolar, 80 N at the first molar, and 100 N at the second molar crown) was applied, which is the case TUB-4. In this case, a different scenario of bone density was observed compared to other cases. In the healthy bone, effective porous and dense implants showed equally reduced bone densities and average Young’s modulus as shown in Fig 11 and Fig 7d.

### 3.5. Quantitative bone density changes in ROIs

**Fig 12:**
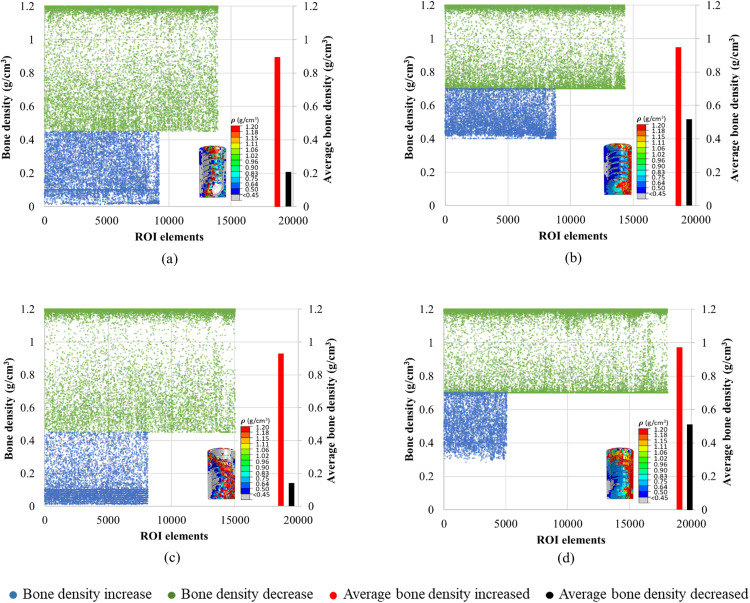
A quantitative bone density distribution in the region of interest (ROI) in the case of TUB-1 in the last iteration; (a) P0.45, E110, (b) P0.7, E110, (c) P0.45, E10, and (d) P0.7, E10.

**Fig 13:**
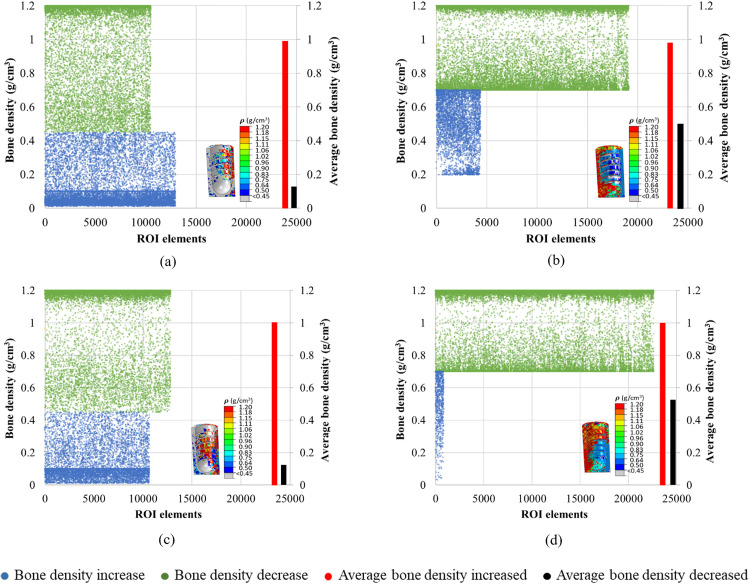
A quantitative bone density distribution in the region of interest (ROI) in the case of TUB-2 in the last iteration; (a) P0.45, E110, (b) P0.7, E110, (c) P0.45, E10, and (d) P0.7, E10.

**Fig 14:**
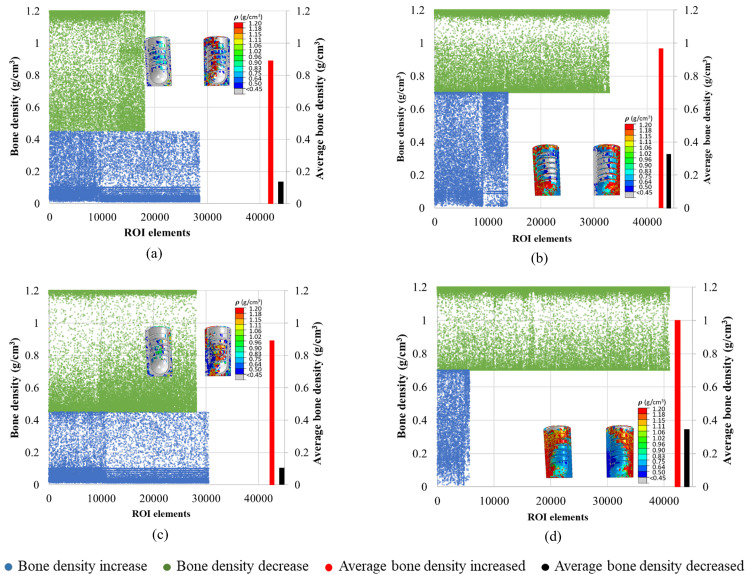
A quantitative bone density distribution in the region of interest (ROI) in the case of TUB-3 in the last iteration; (a) P0.45, E110, (b) P0.7, E110, (c) P0.45, E10, and (d) P0.7, E10.

**Fig 15:**
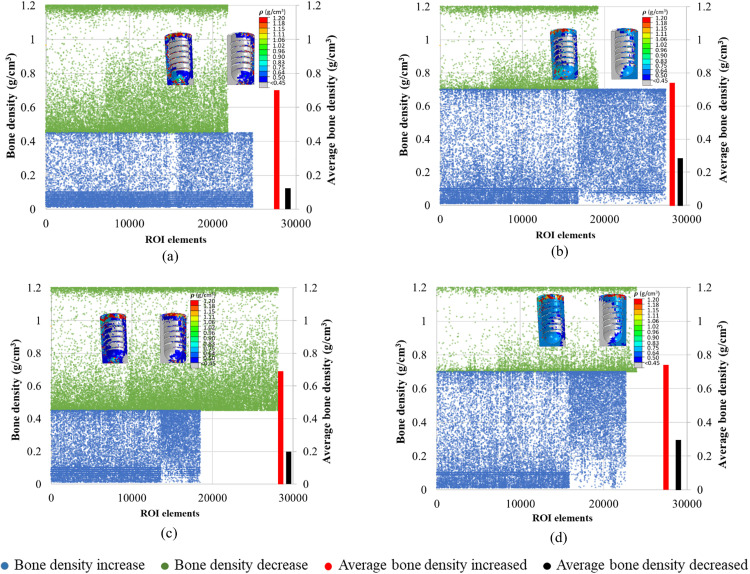
A quantitative bone density distribution in the region of interest (ROI) in the case of TUB-4 in the last iteration; (a) P0.45, E110, (b) P0.7, E110, (c) P0.45, E10, and (d) P0.7, E10.

The quantitative changes in bone density in the last iteration (i.e., when the convergence was achieved) were plotted as illustrated in Figs 12–15. In all cases, a higher number of elements with more elements with bone apposition (increased bone density) and fewer elements with bone resorption (decreased bone density) were observed when effective porous implants (E10) were used. As an illustration, Fig 12 shows the elements of ROI in the case of TUB-1. It was observed that in both instances where the initial bone densities were 0.45 and 0.7 g/cm^3^, the results of an effective porous implant with a stiffness of 10 GPa (E10) showed more ROI elements were falling with increased bone density (green color) compared to the dense implant (E110). Also, the average density of bone in ROIs was lower in the cases of P0.45 and P0.7 with E110 compared to P0.45 and P0.7 with E10. Similarly, Fig 13 shows the changes in elements of ROI in the case of TUB-2. It was seen from the plots that more green elements and fewer blue elements were present in the cases of P0.45, E10, and P0.7, E10 (Fig 13c and Fig 13d) compared to the P0.45, E110, and P0.7, E110 cases in Fig 13a and Fig 13b. Also, higher average bone densities were observed when a lower stiffness implant E10 was simulated with the bone densities 0.45 and 0.7 g/cm^3^. Similar findings were observed in the Figs 14 and 15. These results were also reflected in Figs 8**-**11, but only the location of those elements in ROIs could be seen in those figures. In addition, the quantitative bone density changes plots confirmed that more bone apposition and fewer bone resorption were found in the cases of an effective porous implant (E10) compared to a dense conventional implant (E110).

The results of the simulations can be related to the clinical implications. In clinical studies, bone remodeling (change in bone density) is a major finding across all of implant-based treatments, including implant-supported bridges. A study showed that in fixed partial dentures supported by three or more implants, central implants are more susceptible to marginal bone loss compared to lateral implants, likely due to factors such as limited access for hygiene and prosthetic design [63]. Beyond hygiene challenges, the central implant’s position subjects it to higher biomechanical loads and strain, as it acts as a fulcrum between adjacent implants. This increased mechanical stress can stimulate an adaptive bone remodelling response. While physiological strain promotes bone maintenance, excessive or uneven strain may exceed the bone’s remodelling capacity, leading to localized bone resorption around the implant. Thus, the combined effects of higher mechanical strain and compromised hygiene may synergistically contribute to the greater marginal bone loss observed at central implant sites. Also, mechanical properties and peri-implantitis prevention are considered highly critical perspectives for implant material selection [64]. Moreover, the studies showed the mechanical impact of occlusal overload on the implant and its rehabilitative structure and function. Biologically, load-induced differences in stress distribution around implants can significantly influence both implant micromotion and the response of peri-implant tissues. Unfavorable load transfer can lead to bone resorption, particularly if combined with bacterial insult or inflammatory conditions. High-stress regions may undergo remodelling or micro-damage, creating a local environment that is more susceptible to inflammation and bacterial colonization. This biomechanical stress may therefore contribute to the initiation or progression of peri-implantitis over time [54,65–67]. To overcome the aforementioned complications related to the design of implants, the additive manufacturing technique is now accelerating the research to find the optimal solutions with complex, fascinating implant designs. A study by Mangano et al. [68] showcased the advantages of integrating 3D printing technology within digital dentistry workflows. By combining intraoral scanning, computer-aided design (CAD), and tilting stereolithography (TSLA) 3D printing, the study produced highly precise implant-supported restorations with excellent marginal fit, contact points, and aesthetics. This digital approach streamlines the fabrication process, reduces errors associated with traditional methods, and offers customizable solutions for short-span prostheses. The high survival and low complication rates observed at one year further support the growing role of 3D printing in enhancing the accuracy, efficiency, and clinical success of implant-supported restorations.

### 3.6. Stresses in implants

**Fig 16:**
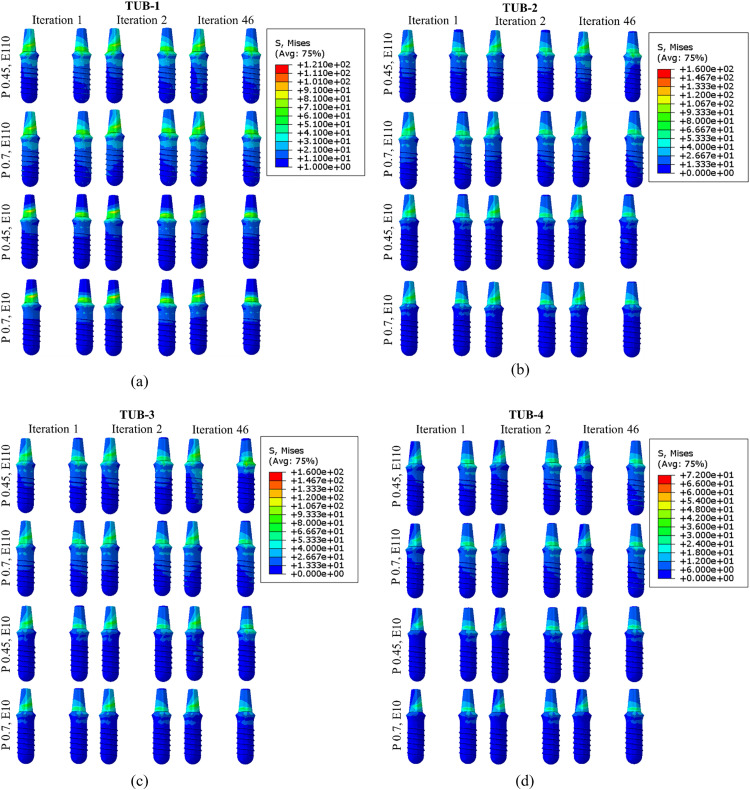
Simulated results of von Mises stresses (MPa) in implants in the cases; (a) TUB-1, (b) TUB-2, (c) TUB-3, and (d) TUB-4.

**Fig 17:**
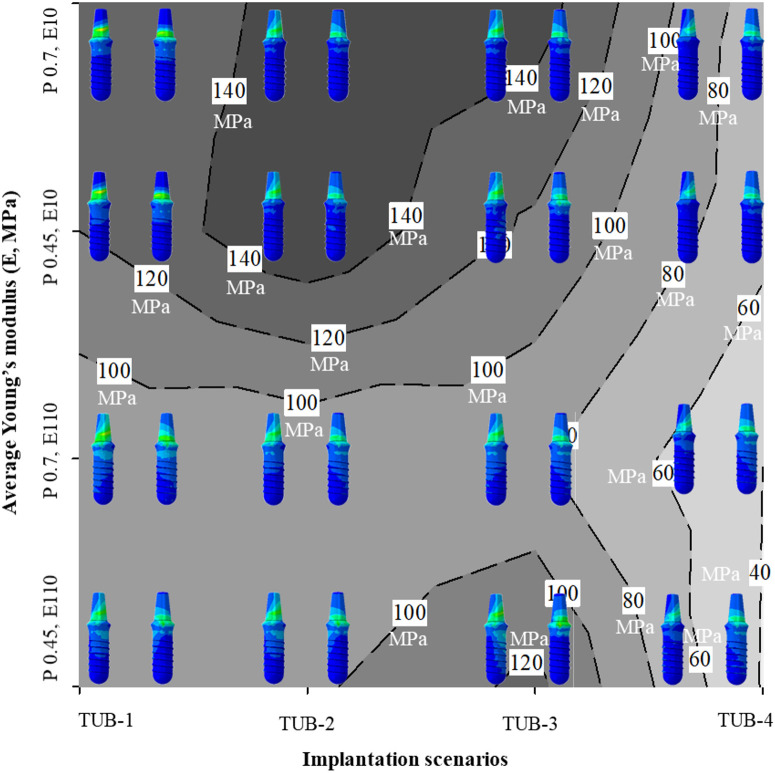
Contour plot showing von Mises stresses (MPa) in all implants in the last iterations of simulations.

The von Mises stresses in the implants for all cases were evaluated and depicted in Fig 16. Fig 16a shows the generated stresses in the implants in the cases of TUB-1 when subjected to the masticating loads. It was observed that in the first iteration in weak bones, 80.6 MPa and 99.9 MPa stresses were generated when dense and effective porous implants were inserted, respectively. Whilst in the healthy bones, 82.8 MPa and 120.6 MPa stresses were generated when dense and effective porous implants were inserted, respectively. However, these stresses were raised when the number of iterations increased and reached a range between 81.44–121.4 MPa. This stress increase reflects the bone density change, which was increased in the ROI with the increase in the number of iterations. The case of TUB-2 (Fig 16b) presented a similar trend. Fig 16c shows the case of TUB-3, where the stresses in implants were decreased during the iteration in the cases of effective porous implants inserted in the weak and healthy bones (156.4 MPa to 115 MPa and from 159.4 MPa to 150.9 MPa for P0.45, E10, and P0.7, E10, respectively). However, the cases of dense implants inserted in weak and healthy bones showed that stress increased from 82.32 to 124.8 MPa and 82.8 MPa to 83.05 MPa, respectively. While the TUB-4 case shown in Fig 16d did not present any noteworthy stress change due to no major changes in the average Young’s moduli and bone density, as previously reported. The results of stresses generated in all the implants in the last iteration are shown in the contour plot as shown in Fig 17. Overall, it was observed that the maximum stresses were generated at the proximal end of the implants in the abutment region [69]. The results show that the highest stresses in the implant were seen in the case of TUB-2 when an effective porous implant was inserted in the healthy bone. Conversely, the lowest stresses were seen in the case of TUB-4 when a dense implant (E110) was inserted in the low bone density (0.45 g/cm^3^). The maximum stress of 160 MPa was shown in the TUB-2 and TUB-3 cases when fixing weak bones with either effective porous or dense implants. However, this stress remained well below the yield stress of commonly used metallic alloys for crown bridge implants, such as Ti-6Al-4V (~880 MPa).

### 3.7. Limitations and recommendations

This study has a few limitations that should be acknowledged. First, the analysis was conducted purely in-silico and should ideally be validated through experimental or in vivo studies. While the implants modeled were treated as having homogeneous material properties, real porous implants exhibit spatially varying stiffness and complex microstructures, which are computationally expensive and beyond the scope of our current study. Similarly, bone tissue was modeled using simplified, linear elastic, and homogeneous properties. In reality, bone is a porous, heterogeneous, and viscoelastic material, and its quality can vary significantly depending on patient-specific factors such as age, overall health, anatomical site, and bone density. Furthermore, boundary conditions such as oral muscle forces and realistic masticatory loading were not considered in this study. Future work should consider multiscale or voxel-based finite element models to incorporate implant and bone porosity more effectively.

## 4. Conclusion

This study examines the biomechanical performance of crown bridge implants fixed in both weak and healthy bone conditions. Dense and porous implants were inserted into the bone, with specific regions of interest defined to analyze changes in bone density using SED based on a bone remodelling algorithm in conjunction with iterative simulations. The results of this study led to the following conclusions.

- In the case of crown bridge implants, when two implants are to be inserted simultaneously, implant stiffness and bone quality are important factors to be considered.
- Porous implants with reduced stiffness exhibited better performance in both regions of interest (ROIs) when only one implant was inserted in healthy and weak bones, provided sufficient time was allowed for bone remodelling before the insertion of the second implant to create a crown bridge.
- If the simultaneous insertion of two implants is necessary, dense implants can be used together in cases of good bone health. However, in weak bone conditions, implant stiffness does not significantly influence the bone remodelling process.
- Loading conditions are critical in the bone remodelling process around surrounding implants when two implants are inserted simultaneously. Poor loading, even in healthy bones, can lead to decreased bone density, with only a slight increase observed in weak bones. However, under poor loading, implant stiffness does not significantly influence bone density changes.

## Supporting information

S1 File Bone density. (XLSX)
